# Assessing support for mental health policies among policy influencers and the general public in Alberta and Manitoba, Canada

**DOI:** 10.1186/s13033-024-00624-y

**Published:** 2024-02-15

**Authors:** Candace I. J. Nykiforuk, Mathew Thomson, Kimberley D. Curtin, Ian Colman, T. Cameron Wild, Elaine Hyshka

**Affiliations:** 1 School of Public Health, University of Alberta, 3-300 ECHA, 11405-87 Ave, Edmonton, AB T6G 1C9 Canada; 2 Clinical Research Coordinator, Clinical Epidemiology Department, Ottawa Hospital Research Institute, 501 Smyth Box 511, Ottawa, ON K1H 8L6 Canada; 3 Department of Emergency Medicine, Faculty of Medicine and Dentistry, University of Alberta, 8303-112 St NW - Room 7-80, Edmonton, AB T6G 2T4 Canada; 4 School of Epidemiology and Public Health, University of Ottawa, 600 Peter Morand Cr – Room 308C, Ottawa, ON K1G 5Z3 Canada; 5 Canada Research Chair in Health System Innovation, School of Public Health, University of Alberta, 3-300 ECHA, 11405-87 Ave, Edmonton, AB T6G 1C9 Canada

**Keywords:** Health policy, Mental health, Public opinion, Knowledge, Attitudes, Beliefs, Nuffield Intervention Ladder, Survey research, Canada, Cumulative link modelling

## Abstract

**Background:**

There is a need to improve mental health policy in Canada to address the growing population burden of mental illness. Understanding support for policy options is critical for advocacy efforts to improve mental health policy. Our purpose was to describe support for population-level healthy public policies to improve mental health among policy influencers and the general public in Alberta and Manitoba; and, identify associations between levels of support and sociodemographic variables and relative to the Nuffield Bioethics Intervention Ladder framework.

**Methods:**

We used data from the 2019 Chronic Disease Prevention Survey, which recruited a representative sample of the general public in Alberta (n = 1792) and Manitoba (n = 1909) and policy influencers in each province (Alberta n = 291, Manitoba n = 129). Level of support was described for 16 policy options using a Likert-style scale for mental health policy options by province, sample type, and sociodemographic variables using ordinal regression modelling. Policy options were coded using the Nuffield Council on Bioethics Intervention Ladder to classify support for policy options by level of intrusiveness.

**Results:**

Policy options were categorized as ‘Provide Information’ and ‘Enable Choice’ according to the Nuffield Intervention Ladder. There was high support for all policy options, and few differences between samples or provinces. Strong support was more common among women and among those who were more politically left (versus center). Immigrants were more likely to strongly support most of the policies. Those who were politically right leaning (versus center) were less likely to support any of the mental health policies. Mental health status, education, and Indigenous identity were also associated with support for some policy options.

**Conclusions:**

There is strong support for mental health policy in Western Canada. Results demonstrate a gap between support and implementation of mental health policy and provide evidence for advocates and policy makers looking to improve the policy landscape in Canada.

## Introduction

In 2016, roughly 16% of the global population suffered from poor mental health in the form of mental or addictive disorders [1]. Furthermore, 7% of the global burden of disease (in disability adjusted life years which combine years of life lost because of disability and premature mortality; DALYs) and 19% of all years lived with disability (YLDs) were caused by mental or addictive disorders [1]. Other scholars have argued that the numbers for the global burden of disease from mental health are even higher (due to stigma and other complex reasons), and suggest that the actual proportion of DALYs and YLDs from mental illness could be almost twice as high due to: overlap in psychiatric and neurological disorders; classifying self-harm and suicide outside of mental illness; conflating all chronic pain with musculoskeletal disorders; exclusion of personality disorders from calculations of mental illness burden; and lack of consideration for mental illness in mortality from associated causes [2]. Importantly, mental health can only be improved if researchers and policymakers account for the inequities in the distribution of poor mental health risk and outcomes [3]. For example, economic disparities within societies such as social class, income inequality, unemployment, houselessness, and poverty are related to the prevalence and incidence of mental health disorders [3–5].

In Canada, one in five residents will experience mental illness of some kind [6]. Besides the high prevalence and the challenges in access to care, the sizable cost of mental illness to the Canadian economy is estimated at $50 billion per year—or 2.8% of Canada’s 2011 gross domestic product (GDP). Without improvements or changes in how Canada approaches mental health, it is estimated that the total cost to the economy will add up to more than $2.5 trillion by 2040 [6, 7]. Despite the universal health care system described by the Canada Health Act [8], there are important subsets of the population who do not access support for mental health or substance use—namely men, older people, members of ethnocultural minorities, newcomers to Canada, those with lower levels of education, and higher income earners [9]. Similar trends were noted in data from 17 countries, where lower-income nations had more unmet need, and importantly use of mental health services was positively related to spending on health care. [10]

In response to these economic, social, and ethical realities, the World Health Organization, among other important institutions, have been highlighting the need for effective mental health policy for years [4, 6, 11]. The previously cited inequities in access to care and support have been argued by researchers and advocates alike to justify legislative and other policy changes that would address these issues, highlighting a need for intervention to address ongoing mental health crises in key demographics, such as students, the elderly, and Indigenous peoples [12–14]. The rolling public health restrictions and need for social distancing due to the COVID-19 pandemic have amplified pre-existing needs for greater access to mental health support [15, 16]. For example, almost 40% of Canadian survey respondents reported deteriorated mental health since COVID-related public health restrictions began, and that individual-focused solutions remain inaccessible, inadequate, or ineffective to most of the general population [16]. The proportional underfunding dedicated to mental health within Canada’s current health budget due to the continued focus on acute care and specialized services demonstrates a lack of prioritization of mental health among policymakers [17]. This trend may be changing, however, with a federal investment of 994.6 million dollars in mental health following the onset of the COVID-19 pandemic [18].

There is a clear need for effective and evidence-informed mental health services systems, yet more supportive policy has not been adopted. Barriers to evidence-based mental health policy include differing perspectives and priorities among advocates; stigma; limited prioritization or perception of need for mental health services among the public and policymakers; and/or economic constraints [19, 20]. Political systems in Canada and the United States also have governments in office for limited terms (i.e., 3- to 5-year election cycles), making longer-term policymaking challenging, particularly in an environment where healthy policy priorities compete for increasingly limited funds [20]. While stigma is a major barrier to the implementation of effective mental health policy, this issue needs to be considered in relation to a prestige hierarchy of mental illnesses, which suggests that the stigma faced by those with mental illnesses is not uniform and some disorders are more accepted or shunned than others [21]. Specifically, the stigma around mood disorders is declining more as individuals view depression more compassionately and with a medical lens [22]. Other disorders like psychosis, schizophrenia, substance use disorders, meanwhile, are still sometimes unfairly associated with criminality or moral failing [21]. In fact, while programs and supports for individuals struggling with personality and psychotic disorders may be supported in principle, they are often met with NIMBYism as individuals and families seek to distance themselves from those struggling with these illnesses, due to largely unfounded fears regarding safety [23–25]. Although the research reviewed here provides indirect evidence for support or opposition of mental health policies, more conclusive evidence on support or opposition to evidence-informed public health policy on mental health is needed to adequately describe the attitudes of Canadians towards mental health policies.

## Rationale and study purpose

In Canada, there is a clear need for more policies built to protect and enhance mental health. In order to see such advancement in our healthcare system, support for these policies must be demonstrated and acted upon by both the general public and policy influencers, like government officials, media outlets, school board members, and large workplaces [26–28]. In addition to those with direct power to enact public policy, several policy actors such as media and the general public have demonstrated effectiveness in influencing policy change through advocacy and awareness raising [29, 30]. Research from Ghana, South Africa, Uganda, and Zambia evidenced how the lack of advocacy from civil society for mental health policy contributed to poor mental health policy implementation [31]. Identifying support or opposition to policy options can expose areas where advocates have made progress in winning support, and where additional targeting may be needed to secure popular and policy maker support for mental health policies. The first purpose of the present study was to describe support for population-level healthy public policies to improve mental health among policy influencers and the general public in Alberta and Manitoba, and further break down levels of support by sociodemographic variables.

We hypothesized that different populations would be more supportive of policies that stand to benefit them most directly. Specifically, we expected Indigenous respondents to support policies aimed at improving First Nation, Inuit, and Métis control over mental health services for their own populations, and immigrant populations to support policies designed to ease the transition into Canadian society. We further hypothesized that respondents with lower self-rated mental health would support policy designed to help them [32]. We also hypothesized that women would be more supportive of the policies because of previous research demonstrating that women are generally more likely to support more intrusive policy options (e.g., beyond providing information or education) and recognize social determinants of health [32–34]. In addition, women are less likely to internalize stigma around mental health, and have lower self-rated health [35, 36]. We expected that political partisanship would be reflected in level of support such that left-leaning voters would be more supportive of most policy options, while right-leaning voters would be more opposed, based on past research [37].

Another factor that can affect support for policy options is the perceived imposition of a policy on personal freedoms [38]. The second aim of this research was to use the Nuffield Council on Bioethics (NCB) Intervention Ladder to classify support for healthy public policies to improve mental health according to the level of individual intrusiveness [39]. The NCB Intervention Ladder provides a framework to identify level of intrusiveness of public health policy initiatives, where each step in the ladder indicates a higher level of state intervention and therefore more restriction on public freedoms [39]. This framework aids in addressing the possible barriers that the infringement on individual liberty may pose to mental health policy expansion [39, 40]. We anticipated that the policies deemed more intrusive (e.g., removing a choice from the general public) and those with more fiscal implications would be the least supported (e.g., enabling choice by building more supervised injection facilities) based on previous studies [38, 41, 42].

## Methods

This study was a secondary analysis of data from the eighth wave of the Chronic Disease Prevention Survey (CDPS) conducted in 2019, which collected responses in Alberta and Manitoba from November 14, 2019 to February 3, 2020. The survey was developed by our research team and piloted using an online platform prior to this data collection period. The CDPS routinely assesses knowledge, attitudes, and beliefs of two groups, policy influencers and the general public, on healthy public policy for population-level chronic disease prevention specific to six key areas: alcohol consumption, tobacco use, healthy eating, physical activity, substance use, and mental health. The questions were randomly ordered within each key area, with one question being presented at a time, and all questions including a “Prefer not to say” option. No incentives were offered for respondents from the policy influencer or general public samples. This study was approved by the University of Alberta Research Ethics Board and all participants completed informed consent prior to starting the survey.

### Participants—general public sample

A random sample of Canadian adults (18 years of age or older; n = 3701) were recruited using a third-party survey firm’s proprietary General Population Random Sample. This sample was comprised of individuals who have previously agreed to be sent survey invitations for public sector studies. Respondents were recruited via phone conversation or voicemail, and then sent a link to the online survey via SMS or email. The target sample size was based on calculations that determined 1537 respondents were needed for a two-sided 95% confidence interval with a width of 0.05 for a sample proportion of 0.5. The survey methods were designed to produce generalizable estimates of public opinion toward mental health policies among adults living in Alberta and Manitoba. Specifically, randomly-drawn panel members residing in these provinces were invited to participate until a quota sample of 3701 respondents matching the age and sex distributions of Canadian adults (18 + years) residing in Alberta and Manitoba was obtained. Data were collected from community-dwelling adults (age 18 +) in Edmonton (n = 639), Calgary (n = 600), Winnipeg (n = 1186), all other municipalities (collectively) in Alberta (n = 553), and all other municipalities (collectively) in Manitoba (n = 723). The overall response rate was 28.3% in Alberta and 23.9% in Manitoba.

### Participants—policy influencer sample

The policy influencer sample included individuals working within three domains of influence: government actors (municipal and provincial), non-governmental leaders (e.g., school board superintendents and human resource managers in large workplaces), and media (e.g., health editors, and editors-in-chief) actors. The research team identified the policy influencer sample by gathering publicly available email addresses for individuals from these domains. The sampling frame was then provided to the third-party survey firm, and respondents received communication from this company on behalf of our research team. These individuals were then emailed a link to complete the survey and received up to five reminder emails. The total final sample size of policy influencer respondents was 420 (Alberta n = 291, Manitoba n = 129), with an overall response rate of 12.5% and 13.7% in Alberta and Manitoba, respectively. Demographic characteristics of these two samples, stratified by province, can be found in Table 1.

**Table 1:** Sociodemographic characteristics of policy influencers and the general public from Alberta and Manitoba respondents to the 2019 Chronic Disease Prevention Survey, n (%)

Sociodemographic characteristics	Alberta		Manitoba	
	General public n = 1648 n (%)	Policy influencers n = 204 n (%)	General public n = 1770 n (%)	Policy influencers n = 98 n (%)
Age (Mean (SD))^b,c,d^	47.67 (16.06)	58.01 (9.94)	48.23 (16.59)	54.90 (10.57)
Gender^b,c^				
Men	799 (48.5)	129 (63.2)	831 (46.9)	47 (48.0)
Women	849 (51.5)	75 (36.8)	939 (53.1)	51 (52.0)
Self-reported physical health				
Excellent	163 (9.9)	22 (10.8)	145 (8.2)	5 (5.1)
Very good	555 (33.7)	66 (32.4)	580 (33.0)	29 (29.6)
Good	591 (35.9)	83 (40.7)	685 (39.0)	43 (43.9)
Fair	264 (16.0)	32 (15.7)	267 (15.2)	19 (19.4)
Poor	73 (4.4)	1 (0.5)	81 (4.6)	2 (2.0)
Self-reported mental health^c^				
Excellent	262 (16.0)	40 (19.6)	272 (15.5)	18 (18.6)
Very good	598 (36.4)	93 (45.6)	614 (35.0)	38 (39.2)
Good	521 (31.7)	57 (27.9)	553 (31.5)	32 (33.0)
Fair	206 (12.5)	11 (5.4)	241 (13.7)	8 (8.2)
Poor	55 (3.3)	3 (1.5)	74 (4.2)	1 (1.0)
Education^a,c,d^				
High school incomplete	34 (2.1)	3 (1.5)	49 (2.8)	0 (0.0)
High school complete	171 (10.5)	13 (6.4)	235 (13.4)	9 (9.2)
University Undergraduate Certificate, Diploma, or Degree	381 (23.3)	33 (16.2)	351 (20.0)	21 (21.4)
University Professional or Graduate Complete	356 (21.8)	97 (47.5)	453 (25.8)	40 (40.8)
College/Technical/University Incomplete	311 (19.0)	24 (11.8)	315 (17.9)	9 (9.2)
College or Technical School Complete	292 (17.8)	27 (13.2)	299 (17.0)	16 (16.3)
Trade School Complete	91 (5.6)	7 (3.4)	53 (3.0)	3 (3.1)
Visible minority identity^c^				
Yes	261 (16.7)	13 (6.5)	287 (17.2)	10 (10.5)
No	1306 (83.3)	186 (93.5)	1384 (82.8)	85 (89.5)
Indigenous identity^a^				
Yes	48 (3.0)	9 (4.5)	121 (7.1)	6 (6.2)
No	1561 (97.0)	190 (95.5)	1593 (92.9)	90 (93.8)
Immigration status^c,d^				
Born in Canada	1340 (81.6)	192 (95.0)	1470 (83.6)	91 (92.9)
Immigrated	302 (18.4)	10 (5.0)	289 (16.4)	7 (7.1)
Gross household income^a,c,d^				
Under $20,000	59 (4.2)	0 (0.0)	79 (5.2)	0 (0.0)
$20,000 to < $40,000	144 (10.2)	6 (3.4)	197 (13.0)	1 (1.2)
$40,000 to < $70,000	271 (19.3)	23 (13.1)	355 (23.4)	21 (24.4)
$70,000 to < $100,000	274 (19.5)	36 (20.5)	342 (22.5)	16 (18.6)
$100,000 to < $125,000	215 (15.3)	18 (10.2)	219 (14.4)	13 (15.1)
$125,000 +	444 (31.6)	93 (52.8)	326 (21.5)	35 (40.7)
Political views^a,b,c^				
Extreme left	43 (2.8)	1 (0.5)	56 (3.4)	1 (1.1)
2	35 (2.3)	2 (1.1)	51 (3.1)	3 (3.4)
3	147 (9.5)	5 (2.7)	211 (12.8)	6 (6.7)
4	201 (13.0)	19 (10.4)	261 (15.8)	12 (13.5)
5	250 (16.1)	32 (17.5)	272 (16.4)	26 (29.2)
6	291 (18.8)	53 (29.0)	301 (18.2)	13 (14.6)
7	224 (14.5)	29 (15.8)	166 (10.0)	10 (11.2)
8	182 (11.7)	31 (16.9)	153 (9.3)	9 (10.1)
9	75 (4.8)	6 (3.3)	87 (5.3)	4 (4.5)
10	35 (2.3)	2 (1.1)	36 (2.2)	4 (4.5)
Extreme right	67 (4.3)	3 (1.6)	60 (3.6)	1 (1.1)

Percent missing for each variable: Age—4.2%; Gender—0%; Self-Reported Physical Health—0.4%; Self-Reported Mental Health—0.6%; Education—0.7%; Visible Minority Identity—5.1%; Indigenous Identity—2.7%; Immigration Status – 0.5%; Household Income—14.3%; Political Views—6.6%

^a^Statistically significant differences between the General Public samples of each province (a = 0.05)

^b^Statistically significant differences between the Policy Influencer samples of each province (a = 0.05)

^c^Statistically significant differences between the Policy Influencer and General Public samples within Alberta (a = 0.05)

^d^Statistically significant differences between the Policy Influencer and General Public samples within Manitoba (a = 0.05)

### Measures

Survey items were developed using a literature review (including authors CIJN and KDC), and further reviewed by both practice and policy experts in the field of mental health (including authors IC, TCW, and EH).

#### Mental health healthy public policy

Respondents indicated their support for 16 healthy public policies (see Table 2) for mental health on a 4-point scale (1 = “Strongly Oppose”, 2 = “Somewhat Oppose”, 3 = “Somewhat Support”, and 4 = “Strongly Support”; participants were also presented with a “Prefer not to say” response option). For the general public sample, who received a subset of 6 questions, the policies were, “Mandate curricula/ training related to mental health promotion, anti-stigma awareness, and suicide prevention among healthcare professionals”, “Implement a school-based prevention programming that incorporates curricula on suicide and related issues (e.g., anxiety-prevention, resiliency-building, socio-emotional health) and expand workshops and peer support programs in schools”, “Provide programs for parents to develop parenting skills and early intervention programs for parents of preschool-aged children”, “Provide information to new immigrants and refugees upon arrival about common mental health problems that may occur with adjustment to Canada and available resources”, “Fund housing services and income supports for individuals with mental health issues”, and “Support First Nations, Métis, and Inuit control of mental health services”. The policy influencer sample received the full survey (16 items) which included the same questions as the general public, as well as, “Subsidize recovery and support programs in shelters to aid in breaking the cycle of family violence”, “Provide maternal mental health resources in all healthcare settings (i.e., trained staff, information for referrals)”, “Fund the development of virtual, technology-based applications to help people access tools, information, and services to address addiction and mental health issues”, “Build or facilitate partnerships across organizations to develop community-service based hubs, which provide a single point of access for multiple social services at one location for families or at-risk population groups (e.g., LGBTQ2S + , newcomers, people with disabilities, veterans…)”, “Legally protect student groups that support the safety and inclusion of marginalized students, including Gay/Straight Alliances as a means of reducing stigma and discrimination in the LGBTQ2S + population”, “Develop and implement inclusive, culturally competent program delivery and training for individuals working in suicide prevention, frontline workers, volunteers, and health care practitioners”, “Promote help-seeking behaviours in men, seniors and other at-risk groups through phone help-lines, reduced individual cost, incentives, and reducing barriers to care”, “Fund media campaigns and targeted education and programming that emphasize the importance of psychological health and safety in the workplace”, “Develop public awareness campaigns against physical and sexual assault”, and “Adapt best practices in suicide prevention used in training healthcare providers in collaboration with First Nations, Métis, and Inuit representatives”.

**Table 2:** Mental Health policy items surveyed with policy influencers (PI) and the general public (GP) and valid percentages of overall support (‘somewhat support’ and ‘strongly support’ combined)

Item	Sample	Shorthand	Overall support (PI)	Overall support (GP)
Mandate curricula/training related to mental health promotion, anti-stigma awareness, and suicide prevention among healthcare professionals	GP/PI	Curricula for health care professionals	96.3%	96.3%
Implement a school-based prevention programming that incorporates curricula on suicide and related issues (e.g., anxiety-prevention, resiliency-building, socio-emotional health) and expand workshops and peer support programs in schools	GP/PI	School-based prevention	95.5%	93.7%
Provide programs for parents to develop parenting skills and early intervention programs for parents of preschool-aged children	GP/PI	Parenting skills programs	96.9%	94.2%
Provide information to new immigrants and refugees upon arrival about common mental health problems that may occur with adjustment to Canada and available resources	GP/PI	New Canadian resources	90.6%	88.9%
Fund housing services and income supports for individuals with mental health issues	GP/PI	Housing and income support	93.1%	89.9%
Support First Nations, Métis, and Inuit control of mental health services	GP/PI	First Nations control of services	86.3%	81.7%
Subsidize recovery and support programs in shelters to aid in breaking the cycle of family violence	PI	Family violence recovery and support	96.6%	n/a
Provide maternal mental health resources in all healthcare settings (i.e., trained staff, information for referrals)	PI	Maternal mental health resources	98.3%	n/a
Fund the development of virtual, technology-based applications to help people access tools, information, and services to address addiction and mental health issues	PI	Mental health technology	94.2%	n/a
Build or facilitate partnerships across organizations to develop community-service based hubs, which provide a single point of access for multiple social services at one location for families or at-risk population groups (e.g., LGBTQ2S + , newcomers, people with disabilities, veterans…)	PI	Community based hubs	91.0%	n/a
Legally protect student groups that support the safety and inclusion of marginalized students, including Gay/Straight Alliances as a means of reducing stigma and discrimination in the LGBTQ2S + population	PI	Protect 2SLGBTQ + students	83.4%	n/a
Develop and implement inclusive, culturally competent program delivery and training for individuals working in suicide prevention, frontline workers, volunteers, and health care practitioners	PI	Inclusive program delivery	97.2%	n/a
Promote help-seeking behaviours in men, seniors and other at-risk groups through phone help-lines, reduced individual cost, incentives, and reducing barriers to care	PI	At-risk help-seeking facilitation	98.0%	n/a
Fund media campaigns and targeted education and programming that emphasize the importance of psychological health and safety in the workplace	PI	Workplace psychological safety	92.3%	n/a
Develop public awareness campaigns against physical and sexual assault	PI	Assault public awareness	97.5%	n/a
Adapt best practices in suicide prevention used in training healthcare providers in collaboration with First Nations, Métis, and Inuit representatives	PI	Suicide prevention in First Nations	94.1%	n/a

#### Nuffield council on bioethics intervention ladder coding

To examine whether the intrusiveness of a policy may be related to the level of either public or policy influencer support, we used the NCB Intervention Ladder as a framework [39]. The ladder levels, by increasing intrusiveness, are (1) do nothing or simply monitor the current situation, (2) provide information, (3) enable choice, (4) guide choices through changing the default policy, (5) guide choices through incentives, (6) guide choices through disincentives, (7) restrict choice, and 8) eliminate choice. This ethical framework for public health argues that more intrusive interventions require stronger justifications, balancing the benefits of collective action against losses to individual liberty [39]. By examining support for the mental health policies within this framework, it may provide a deeper understanding of why certain policies garnered more or less support.

Two research assistants coded the policy questions with the NCB Intervention Ladder, using a codebook developed by our team to ensure consistency [43]. This codebook was developed to address what has been described as limited, and sometimes conflicting reports on the interpretation of each rung of the NCB Intervention Ladder [44]. During coding, we focused on how policies would affect the liberties of the “general public” (i.e., the freedom of lay-individuals) rather than impacts to government or industry. The two coders met to discuss their coding after the first round, and arrived at the final codes via consensus. The mean percentage of respondent support at each level of the Likert scale were compared using paired, two-sided t-tests with an alpha of 0.05 to assess for differences.

#### Sociodemographic variables

*Age* We assessed age by asking “How old are you today?” recorded as a number between 18 and 120. These values were kept continuous for analyses.

*Gender* Gender was assessed by asking, “How would you describe your current gender?”, with the options being “Man”, “Woman”, “Gender diverse”, or “Other”, which gave the option to specify. Because of a very small number of gender diverse and other respondents, these observations were removed to create a binary category that preserved sample size and degrees of freedom, which is one limitation of this study.

*Self-reported physical health status* Assessment of self-reported physical health was done by asking “In general, would you say your physical health is excellent, very good, good, fair or poor?” on a five-point scale.

*Self-reported mental health status* Assessment of self-reported mental health was done by asking “In, general would you say your mental health is excellent, very good, good, fair or poor?” on a five-point scale.

*Educational attainment* We assessed educational attainment by asking “What is the highest level of education you have completed?”. Participants then selected from a list of: “Did not complete high school”, “High school”, “Trade school”, “Some college, technical school, or university”, “College or technical school”, “University undergraduate certificate, diploma, or degree”, or “University graduate or professional degree”.

*Visible minority identity* Respondents were asked, “Do you consider yourself to be a member of a visible minority?” with possible answers being “Yes” or “No”.

*Indigenous identity* We assessed whether or not a respondent identified as Indigenous, Aboriginal, First Nations, or Métis by asking “Do you identify yourself as Indigenous, Aboriginal, First Nations or Métis?” with possible answers being “Yes” or “No”.

*Immigration Status* Respondents chose from two options: “Born in Canada”, or “Moved to Canada from somewhere else”.

*Gross annual household income* We assessed annual household income by asking “Which of the following categories best describes the TOTAL income of ALL members of your household for the past year, BEFORE taxes and deductions?” Participants then selected from a list of potential income ranges: “40,000 to just under $70,000”, “$70,000 to just under $100,000”, “$100,000 to just under $125,000”, or “$125,000 or more”.

*Political views* Respondents were asked “In politics, people sometimes talk of ‘left/liberal’ and ‘right/conservative’. Where would you place yourself on a scale from 1 to 11, where 1 means extreme left and 11 means extreme right?”. Options were kept as ordinal categories from “1” to “11”.

### Data analysis

#### Missing data and imputation

All data analyses were completed using R version 3.6.0 using the RStudio IDE [45]. A small percentage (380 of the 4100 total observations; 9%) were removed because all questions were missing (either No response, Prefer not to say, or left blank). Analysis of the remaining observations showed that 5% or less were missing for all sociodemographic variables except for income (14.4% in general public, 13.3% for policy influencers). Missingness of policy questions was similarly low, with 5% or less missing. Given that inspection of the data indicated few patterns, we assumed that the data were missing at random and thus suitable for multiple imputation [46]. Multiple imputation was done using the multivariate imputation by chained equations method via the *mice* package, using predictive mean matching for age, logistic regression for binary variables, polytomous logistic regression for unordered categorical variables (n > 2 categories), and proportional odds modeling for ordered categorical variables. This process used 25 iterations and 30 imputations, using more iterations and two-times the percent of income data that was missing as a guide to be more conservative. Probabilities for all models were produced in accordance with Rubin’s rules, with models being fitted on each imputed data set separately and predictive probabilities then averaged across them to produce final estimates.

#### Variable selection and modeling

In order to examine differences across the four Likert levels of each question while respecting the unique constructs addressed in each question, an ordinal regression procedure was run using cumulative link models built separately for each item. This method was selected as it allows for examining differences across all categories, and preserves more information than collapsing into simple agree/disagree categories. Explanatory modelling relies heavily on subject matter and other a priori knowledge. Because this is a novel area of policy analysis, however, there is a dearth of literature and established evidence. As such, modelling relied on more data-driven approaches. Using a Bayesian approach, this involved examining the posterior probability that each socio-demographic variable is non-zero in the regression equation, systematically removing one explanatory variable at a time while controlling for remaining variables in the complete models, and manually examining all model possibilities for changes in coefficients. Regression coefficients were transformed from the log scale into odds ratio estimates with 95% confidence intervals, and Holm's Sequential Bonferroni Procedure was used to adjust for multiple testing. Imputation was only run on items asked to both samples because the policy influencer sample size is too small and unstable for this procedure. Validity of imputations were assessed by visual examination of imputation data and strip plots, and the proportional odds assumption for the models was assessed using graphical methods as described by Harrell [47]. The following packages were used to complete the analyses in R: *tidyr, plyr, ggplot2, foreign, dplyr, mice, Hmisc, tableone, naniar, BMA, MASS, reshape2, MPDiR, jtools, lme4,* and *ordinal*.

## Results

Overall, the majority of respondents in both provinces and sample populations were either strongly or somewhat supportive of all policies about which they were asked. For almost all policies, 50% of respondents were strongly supportive. The two most popular policies among the general public were “Mandate curricula/ training related to mental health promotion, anti-stigma awareness, and suicide prevention among healthcare professionals” (96.7% support) and “Provide programs for parents to develop parenting skills and early intervention programs for parents of preschool-aged children” (94.2% support). For policy influencers, the most popular options were “Provide maternal mental health resources in all healthcare settings (i.e., trained staff, information for referrals)” (98.5% support) and “Develop and implement inclusive, culturally competent program delivery and training for individuals working in suicide prevention, frontline workers, volunteers, and health care practitioners” (98.2% support). In contrast, the most opposed policies among the general public were “Provide information to new immigrants and refugees upon arrival about common mental health problems that may occur with adjustment to Canada and available resources” (10.9% opposed) and “Support First Nations, Métis, and Inuit control of mental health services” (18.0% opposed). Among policy influencers, the most opposed policies were “Legally protect student groups that support the safety and inclusion of marginalized students, including Gay/Straight Alliances as a means of reducing stigma and discrimination in the LGBTQ2S + population” (12.3% oppose) and “Support First Nations, Métis, and Inuit control of mental health services” (11.8% oppose). A full overview of survey responses, and support and opposition for the 16 healthy public policies can be found in Table 3.

**Table 3:** Proportion of support and opposition responses for mental health policy options grouped by modified Nuffield Council on Bioethics Intervention Ladder categories for policy influencers and the General Public in the 2019 Chronic Disease Prevention Survey, n (%)

Nuffield Intervention Ladder category	Policy and level of support Strongly oppose: StO Somewhat oppose: SoO Somewhat support: SoS Strongly support: StS	Alberta		Manitoba	
		General public n = 1648	Policy influencers n = 204	General public n = 1770	Policy influencers n = 98
1 – Provide Information	Provide information to new immigrants and refugees upon arrival about common mental health problems that may occur with adjustment to Canada and available resources (Q55.10)^a^	StO: 82 (5.2) SoO: 109 (6.8) SoS: 553 (34.7) StS: 848 (53.3)	9 (4.8) 9 (4.8) 82 (43.9) 87 (46.5)	62 (3.6) 106 (6.2) 550 (32.0) 1000 (58.2)	3 (3.2) 4 (4.3) 33 (35.1) 54 (57.4)
	Fund media campaigns and targeted education and programming that emphasize the importance of psychological health and safety in the workplace (Q55.13)	N/A	StO: 7 (3.5) SoO: 8 (4.0) SoS: 85 (42.9) StS: 98 (49.5)	N/A	1 (1.1) 5 (5.4) 34 (36.6) 53 (57.0)
	Develop public awareness campaigns against physical and sexual assault (Q55.14)	N/A	StO: 0 (0.0) SoO: 4 (2.0) SoS: 61 (30.5) StS: 135 (67.5)	N/A	1 (1.0) 2 (2.1) 21 (21.6) 73 (75.3)
2 – Enable Choice	Mandate curricula/training related to mental health promotion, anti-stigma awareness, and suicide prevention among healthcare professionals (Q55.1)	StO: 21 (1.3) SoO: 31 (1.9) SoS: 447 (27.7) StS: 1112 (69.0)	0 (0.0) 7 (3.5) 66 (33.2) 126 (63.3)	14 (0.8) 46 (2.6) 433 (24.9) 1247 (71.7)	1 (1.1) 2 (2.1) 27 (28.4) 65 (68.4)
	Implement a school-based prevention programming that incorporates curricula on suicide and related issues (e.g., anxiety-prevention, resiliency-building, socio-emotional health) and expand workshops and peer support programs in schools (Q55.2)	StO: 32 (2.0) SoO: 74 (4.6) SoS: 450 (28.2) StS: 1042 (65.2)	1 (0.5) 9 (4.5) 66 (33.0) 124 (62.0)	26 (1.5) 71 (4.1) 491 (28.5) 1136 (65.9)	2 (2.1) 1 (1.0) 29 (29.9) 65 (67.0)
	Subsidize recovery and support programs in shelters to aid in breaking the cycle of family violence (Q55.3)	N/A	StO: 1 (0.5) SoO: 5 (2.5) SoS: 61 (30.7) StS: 132 (66.3)	N/A	1 (1.0) 3 (3.1) 26 (27.1) 66 (68.8)
	Provide maternal mental health resources in all healthcare settings (i.e., trained staff, information for referrals) (Q55.4)	N/A	StO: 0 (0.0) SoO: 2 (1.0) SoS: 73 (37.1) StS: 122 (61.9)	N/A	0 (0.0) 2 (2.1) 32 (33.7) 61 (64.2)
	Provide programs for parents to develop parenting skills and early intervention programs for parents of preschool-aged children (Q55.5)	StO: 29 (1.8) SoO: 70 (4.4) SoS: 524 (32.8) StS: 975 (61.0)	0 (0.0) 3 (1.5) 71 (35.5) 126 (63.0)	23 (1.3) 71 (4.1) 519 (30.1) 1113 (64.5)	1 (1.1) 3 (3.2) 29 (30.5) 62 (65.3)
	Fund the development of virtual, technology-based applications to help people access tools, information, and services to address addiction and mental health issues (Q55.6)	N/A	StO: 2 (1.0) SoO: 9 (4.7) SoS: 74 (38.3) StS: 108 (56.0)	N/A	1 (1.1) 5 (5.5) 37 (40.7) 48 (52.7)
	Build or facilitate partnerships across organizations to develop community-service based hubs, which provide a single point of access for multiple social services at one location for families or at-risk population groups (e.g., LGBTQS2 + , newcomers, people with disabilities, veterans…) (Q55.7)	N/A	StO: 8 (4.1) SoO: 12 (6.2) SoS: 75 (38.9) StS: 98 (50.8)	N/A	3 (3.3) 2 (2.2) 34 (37.4) 52 (57.1)
	Legally protect student groups that support the safety and inclusion of marginalized students, including Gay/Straight Alliances as a means of reducing stigma and discrimination in the LGBTQ2S + population (Q55.8)	N/A	StO: 15 (8.1) SoO: 18 (9.7) SoS: 47 (25.4) StS: 105 (56.8)	N/A	4 (4.5) 2 (2.2) 23 (25.8) 60 (67.4)
	Develop and implement inclusive, culturally competent program delivery and training for individuals working in suicide prevention, frontline workers, volunteers, and health care practitioners (Q55.9)	N/A	StO: 2 (1.0) SoO: 3 (1.5) SoS: 60 (30.2) StS: 134 (67.3)	N/A	0 (0.0) 1 (1.1) 23 (24.2) 71 (74.7)
	Fund housing services and income supports for individuals with mental health issues (Q55.11)^a^	StO: 37 (2.3) SoO: 134 (8.4) SoS: 667 (41.8) StS: 757 (47.5)	3 (1.5) 11 (5.6) 87 (44.6) 94 (48.2)	40 (2.3) 117 (6.8) 641 (37.4) 916 (53.4)	2 (2.1) 2 (2.1) 38 (40.4) 52 (55.3)
	Promote help-seeking behaviours in men, seniors and other at-risk groups through phone help-lines, reduced individual cost, incentives, and reducing barriers to care (Q55.12)	N/A	StO: 0 (0.0) SoO: 4 (2.1) SoS: 75 (38.5) StS: 116 (59.5)	N/A	1 (1.1) 1 (1.1) 34 (37.0) 56 (60.9)
	Support First Nations, Métis, and Inuit control of mental health services (Q55.15)^b^	StO: 122 (7.9) SoO: 160 (10.3) SoS: 509 (32.8) StS: 760 (49.0)	13 (6.7) 18 (9.3) 57 (29.4) 106 (54.6)	139 (8.3) 159 (9.5) 501 (30.1) 868 (52.1)	3 (3.2) 4 (4.3) 39 (41.5) 48 (51.1)
	Adapt best practices in suicide prevention used in training healthcare providers in collaboration with First Nations, Métis, and Inuit representatives (Q55.16)	N/A	StO: 6 (3.1) SoO: 7 (3.6) SoS: 60 (30.6) StS: 123 (62.8)	N/A	2 (2.1) 2 (2.1) 24 (25.0) 68 (70.8)

No statistically significant (a = 0.05) differences in support were found between the policy influencers of the two provinces, or between the policy influencer and general public samples in Alberta

Percent missing for each variable: Q55.10–3.5%; Q55.13–3.6%; Q55.14–1.7%; Q55.1–2.0%; Q55.2–2.7%; Q55.3–2.3%; Q55.4–3.3%; Q55.5–2.7%; Q55.6–6.0%; Q55.7–6.0%; Q55.8–9.3%; Q55.9–2.6%; Q55.11–3.3%; Q55.12–5.0%; Q55.15–5.8%; Q55.16–3.3%

^a^Indicates statistically significant differences between the General Public samples of each province (a = 0.05)

^b^Indicates statistically significant differences between the Policy Influencer and General Public samples within Manitoba (a = 0.05)

### Cumulative link models

The results of the cumulative link models can be found in Table 4. To conserve space, the odds ratios and confidence intervals are reported only for province, sample, and covariates that were found to be significant at the 0.05 level after applying a Holm Bonferroni correction. Graphical methods as well as likelihood ratio tests of the proportional odds assumption were used to determine whether variables included in the models should be added as nominal effects to preserve the validity of the proportional odds assumption [47]. Due to the low number of respondents who identified as Indigenous or who identified as being politically far-left or far-right, it was not always possible to visualize the cut-points for these variables, and likelihood ratio tests were used instead.

**Table 4:** Results of the ordinal regression on support for mental health policy options by sociodemographic variables in the 2019 Chronic Disease Prevention Survey

Policy	Model covariates	Nominal effects	Odds ratios for province and sample type (95% confidence interval)	Significant variables	Odds ratio (95% confidence interval)
Mandate curricula/ training related to mental health promotion, anti-stigma awareness, and suicide prevention among healthcare professionals (Q55.1)	Age, Gender, Mental Health, Immigration Status, Indigenous Identity, Political Alignment, Sample	Visible Minority Status, Province	Manitoba: N/A Policy Influencer: 1.06 (0.81–1.39)	Gender (vs. man) Mental Health (vs. excellent) Immigrant (vs. no) Political Alignment (vs. centre (6))	Gender—Woman 2.33 (2.00–2.71) Mental Health—Fair 1.64 (1.21–2.21) Mental Health—Poor 2.36 (1.36–4.10) Immigrant—Yes 1.53 (1.22–1.92) Political Alignment – Extreme Left 4.06 (2.06–7.99) Political Alignment—2 4.33 (2.03–9.22) Political Alignment—3 3.32 (2.33–4.74) Political Alignment—4 2.12 (1.60–2.80) Political Alignment—5 1.52 (1.18–1.96) Political Alignment—8 0.57 (0.44–0.74) Political Alignment – 10 0.42 (0.26–0.68)
Implement a school-based prevention programming that incorporates curricula on suicide and related issues (e.g., anxiety-prevention, resiliency-building, socio-emotional health) and expand workshops and peer support programs in schools (Q55.2)	Gender, Mental Health, Immigration Status, Political Alignment, Province, Sample	Visible Minority Status	Manitoba: 0.96 (0.83–1.11) Policy Influencer: 1.30 (1.00–1.68)	Gender (vs. man) Mental Health (vs. excellent) Immigrant (vs. no) Political Alignment (vs. centre (6))	Gender—Woman 2.08 (1.80–2.41) Mental Health—Fair 1.81 (1.37–2.39) Mental Health—Poor 2.24 (1.39–3.61) Immigrant—Yes 1.42 (1.14–1.77) Political Alignment – Extreme Left 3.78 (2.06–6.94) Political Alignment—2 3.35 (1.77–6.35) Political Alignment—3 2.19 (1.62–2.95) Political Alignment—4 1.95 (1.49–2.55) Political Alignment—8 0.57 (0.44–0.75) Political Alignment—9 0.55 (0.39–0.77) Political Alignment – 10 0.37 (0.23–0.60)
Provide programs for parents to develop parenting skills and early intervention programs for parents of preschool-aged children (Q55.5)	Age, Gender, Immigration Status, Indigenous Identity, Political Alignment, Province, Sample	N/A	Manitoba: 1.03 (0.90–1.19) Policy Influencer: 1.48 (1.14–1.91)	Age Gender (vs. man) Immigrant (vs. no) Political Alignment (vs. centre (6))	0.99 (0.99–1.00) Gender—Woman 1.90 (1.65–2.18) Immigrant—Yes 1.47 (1.21–1.79) Political Alignment—Extreme Left 5.30 (2.65–10.60) Political Alignment—2 3.29 (1.78–6.11) Political Alignment—3 2.17 (1.60–2.93) Political Alignment—4 1.71 (1.33–2.21) Political Alignment—5 1.39 (1.10–1.77) Political Alignment—7 0.68 (0.53–0.87) Political Alignment—8 0.55 (0.43–0.71) Political Alignment—9 0.50 (0.35–0.70) Political Alignment—10 0.44 (0.27–0.71)
Provide information to new immigrants and refugees upon arrival about common mental health problems that may occur with adjustment to Canada and available resources (Q55.10)	Gender, Education, Immigration Status, Political Alignment, Province, Sample	Age	Manitoba: 1.10 (0.96–1.26) Policy Influencer: 0.98 (0.76–1.25)	Gender (vs. man) Education (vs. University Professional or Graduate Complete) Immigrant (vs. no) Political Alignment (vs. centre (6))	Gender – Woman 1.54 (1.34–1.77) Education—High School Complete 0.70 (0.54–0.89) Education—College/Technical/University Incomplete 0.67 (0.54–0.83) Education—Trade School Complete 0.43 (0.30–0.61) Immigrant—Yes 1.81 (1.49–2.21) Political Alignment—Extreme Left 4.59 (2.66–7.92) Political Alignment—2 3.87 (2.18–6.87) Political Alignment—3 3.66 (2.70–4.95) Political Alignment—4 2.71 (2.10–3.49) Political Alignment—5 1.75 (1.40–2.19) Political Alignment—7 0.70 (0.55–0.88) Political Alignment—8 0.48 (0.38 -0.62) Political Alignment—9 0.45 (0.32–0.64) Political Alignment—Extreme Right 0.33 (0.22–0.49)
Fund housing services and income supports for individuals with mental health issues (Q55.11)	Gender, Education, Indigenous Identity, Immigration Status, Political Alignment, Province, Sample	Age	Manitoba: 1.09 (0.95–1.24) Policy Influencer: 1.26 (0.98–1.63)	Gender (vs. man) Education (vs. University Professional or Graduate Complete) Political Alignment (vs. centre (6))	Gender—Woman 1.44 (1.26–1.66) Education—Trade School Complete 0.56 (0.40–0.80) Political Alignment—Extreme Left 8.19 (4.19–16.02) Political Alignment—2 5.17 (2.89–9.24) Political Alignment—3 3.19 (2.40–4.23) Political Alignment—4 2.36 (1.85–3.02) Political Alignment—7 0.59 (0.47–0.75) Political Alignment—8 0.47 (0.37–0.60) Political Alignment—9 0.39 (0.28–0.55)
Support First Nations, Métis, and Inuit control of mental health services (Q55.15)	Age, Gender, Indigenous Identity, Immigration Status, Household Income, Political Alignment, Province, Sample	Mental Health	Manitoba: 0.89 (0.78–1.01) Policy Influencer: 1.78 (1.38–2.28)	Gender (vs. man) Indigenous Identity (vs. no) Immigration Status (vs. no) Political Alignment (vs. centre (6))	Gender – Woman 1.74 (1.52–1.98) Indigenous Identity—Yes 2.23 (1.58–3.15) Immigration Status—Yes 1.75 (1.46–2.11) Political Alignment—Extreme Left 3.01 (1.82–4.98) Political Alignment—2 5.33 (2.86–9.95) Political Alignment—3 3.24 (2.45–4.30) Political Alignment—4 2.13 (1.67–2.72) Political Alignment—7 0.54 (0.43–0.68) Political Alignment—8 0.44 (0.35–0.56) Political Alignment—9 0.44 (0.32–0.60) Political Alignment—10 0.47 (0.30–0.72) Political Alignment—Extreme Right 0.50 (0.34–0.73)

There were no differences in the odds of supporting any of the policies by province. Policy influencers were more likely to strongly support (versus somewhat support or somewhat oppose) programs for parents to learn skills compared to the general public (OR: 1.48, 95% CI 1.14–1.91), and strongly support (versus somewhat support or somewhat oppose) First Nations, Métis, and Inuit control of mental health services (OR: 1.78, 95% CI 1.38–2.28), but no other differences were found between policy influencers and the general public. For all of the policies analysed, more strong support was likely in women (versus men; OR range: 1.44 [95% CI 1.26–1.66]–2.33 [95% CI 2.00–2.71]), and those who were more politically left (versus center; OR range: 1.39 [95% CI 1.10–1.77]–8.19 [95% CI 4.19–16.02]). Immigrants were more likely to strongly support all the policies (versus non-immigrants; OR range: 1.42 [95% CI 1.14–1.77]–1.81 [95% CI 1.49–2.21]) except for “Fund housing services and income supports for individuals with mental health issues”. Those who were politically right leaning (versus center) were less likely to support any of the mental health policies (OR range: 0.33 [95% CI 0.22–0.49]–0.70 [95% CI 0.55–0.88]).

In the model for the policy “Mandate curricula/ training related to mental health promotion, anti-stigma awareness, and suicide prevention among healthcare professionals”, those with fair or poor mental health (versus excellent), were more likely to support this policy (fair vs. excellent—OR: 1.64, 95% CI 1.21–2.21; poor vs. excellent—OR: 2.36, 95% CI 1.36–4.10). The model for “Implement a school-based prevention programming that incorporates curricula on suicide and related issues (e.g., anxiety-prevention, resiliency-building, socio-emotional health) and expand workshops and peer support programs in schools” showed that those with fair or poor mental health (versus excellent mental health (fair versus excellent – OR: 1.81, 95% CI 1.37–2.39); poor vs. excellent—OR: 2.24, 95% CI 1.39–3.61) were more supportive.

For the policy “Provide programs for parents to develop parenting skills and early intervention programs for parents of preschool-aged children”, higher age decreased odds of strongly supporting the policy (per 1 year older—OR: 0.99, 95% CI 0.99–1.00). The policy “Provide information to new immigrants and refugees upon arrival about common mental health problems that may occur with adjustment to Canada and available resources” was less likely to be supported by those who completed high school (versus university professional or graduate complete—OR: 0.70, 95% CI 0.54–0.89); those with incomplete college/technical/ university (versus university professional or graduate complete—OR: 0.67, 95% CI 0.54–0.83); or those who completed trade school (versus university professional or graduate complete—OR: 0.43, 95% CI 0.30–0.61). Education was also related to “Fund housing services and income supports for individuals with mental health issues”, where less support was likely for those who completed trade school (versus university professional or graduate complete—OR: 0.56, 95% CI 0.40–0.80). Lastly, the model for “Support First Nations, Métis, and Inuit control of mental health services” was more likely to be supported by those with an Indigenous identity (versus no Indigenous identity—OR: 2.23, 95% CI 1.58–3.15).

### Nuffield council on bioethics intervention ladder coding

Results of the NCB Intervention Ladder coding also can be found in Table 4, along with the percent of respondents who responded at each Likert level for each question, stratified by province and sample type. This table shows that all of the policy options were characterized as either Provide Information or Enable Choice. There was no difference in support for policies between Provide Information (Strongly Support M = 59.10%, Somewhat Support M = 34.14%, Somewhat Oppose M = 4.28%, Strongly Oppose M = 2.48%) and Enable Choice (Strongly Support M = 60.96%, Somewhat Support M = 32.44%, Somewhat Oppose M = 4.24%, Strongly Oppose M = 2.35%). In the general public samples, Manitoba was more “strongly supportive” (compared to Alberta) of the policies “Fund housing services and income supports for individuals with mental health issues” and “Provide information to new immigrants and refugees upon arrival about common mental health problems that may occur with adjustment to Canada and available resources”. Policy influencers in Manitoba were more supportive of “Support First Nations, Métis, and Inuit control of mental health services” compared to the general public in Manitoba. There were no significant differences between the policy influencer samples of the two provinces, nor between policy influencers and the general public in Alberta.

## Discussion

This study evaluated support for mental health policies among the general public and policy influencers in Alberta and Manitoba, Canada to describe the appetite for mental health policy. To our knowledge this is the first study to measure mental health policy support. We also examined support by sociodemographic variables, and levels of the NCB Intervention Ladder [39]. Overall, there was strong support across all policy options, which could be reflective of the relatively low intrusiveness of all the policy options as described by the NCB Intervention Ladder (categorized as Provide Information and Enable Choice) [38, 39]. Differences between general public and policy influencer samples were few and were not statistically significant in the models controlling for covariates. This alignment is contrary to the notion that the barrier to policy implementation is public or political opposition [19, 20]. Another reason for the high support may be social desirability in responding and the general understanding that these policies likely have positive effects. Note, we did not ask respondents to rank options relative to other policy priorities such as low taxes, education, healthcare, etc.

The high amount of support demonstrates a disconnection between supporting potentially helpful policies and their implementation. Indeed, strong support for these policies is necessary, but insufficient to assume they are high priorities among policy influencers or the general public. Policies with strong empirical support, like housing and income supports [48, 49] were just as strongly supported as some that are less resource intensive and generally less effective policies like informational campaigns [50, 51]. For example, the recent Alberta budget did not include funding for supportive housing efforts with mental health and addiction support in the capital city of Edmonton despite a specific request from the Mayor [52]. Housing and income supports are more expensive and potentially a more contentious policy option due to NIMBYism [24]. Particularly, as housing services are made available to those with mental health issues, neighbourhood associations and even individuals may complain of a perceived reduction in the safety of their community and in real estate values while still espousing support for these kinds of housing supports [53]. Advocates should continue to promote effective, evidence-based policy options for the most impactful systems changes and target common misconceptions about programs like housing supports.

These results reflect the dominant paradigm in mental health which is centered on individual treatment, usually using pharmaceuticals, rather than promotion or prevention [54]. The cultural focus on the individual, while contrary to the recommendations of World Health Organization policy instruments [11], does not lend itself well to the drafting of population-level mental health legislation. The focus on more individual level solutions is demonstrated in the selection of mental health policy options that were included in the CDPS, which were drawn from extant literature and a scan of recommended current and policies in practices in Canada and vetted by mental health practitioners and researchers as potentially acceptable and viable in a provincial environment. In addition to the current perceived options for improving public mental health, scholars have noted reasons that public mental health policy implementation often fails within top-down implementation partnerships including lack of understanding and agreement of what mental health promotion is among key players, under identification of stakeholders, partnership difficulties, scattered responsibility for implementation, trust and personal relationship issues, and poor engagement with vulnerable groups [55]. An exploration of more population-level approaches to mental health support and prevention as well as implementation strategies would be a valuable extension of this work.

### Sample and sociodemographic differences in support

The cumulative link modelling did not show any differences in support between provinces, and differences in support between the general public and policy influencer samples were minimal. Importantly, this broad-based support for a range of mental health policy options could lend much needed evidence for advocacy efforts to advance policy [56, 57]. The Alberta branch of the Canadian Mental Health Association specified several areas of improvement related to the policies examined in the CDPS and stemming from the Valuing Mental Health Report developed in 2015 and updated in 2017 [58, 59]. Namely, increasing spending from 6 to 13% of the total health budget; coordination of primary care, clinical care, and community service delivery; prioritizing interventions for youth and seniors; generating long term, affordable supportive housing for those living with mental illness and support/ education for family or peers; and greater cross-ministry involvement in Indigenous focussed services. Similarly, Manitoba was called to invest 9.2% of its health care spending in mental health and addictions in their 2020 budget, ensuring services were not concentrated within their capital region, and increasing focus on newcomer mental health supports [60]. Both provinces vowed to increase spending and improve mental health and addictions services following the collection of the data used in this study [61, 62].

Only two models showed significant differences between the samples. Policy influencers were more likely to support policies that touched on supporting parents and children and on giving more control to First Nations, Métis, and Inuit people with regard to mental health services. These policies may generate positive optics for policy influencers of better serving children and families, or their greater awareness of societal responsibility for action on population-level determinants of health. Future research examining the values and priorities underpinning the support for respective policy options could reveal further insights into the drivers of these differences.

Across all the policy options assessed, we found that women (versus men), and left–leaning voters (versus center) were more likely to support all the policies, while right-leaning (versus center) were less likely to support any of the policies. Immigrants to Canada (versus non-immigrants) were more likely to support all the policies except funding housing and income supports. Other variables like education, mental health, and Indigenous identification were also significant for some of the models.

As we hypothesized, women had 1.5–2.3 times the odds of strongly supporting a policy (versus somewhat supporting or somewhat opposing) when compared to men, all other covariates being held equal. This is not surprising given the research showing that men are far less likely to seek help for mental health problems [63]; express mental distress differently than women [64, 65]; are more likely to internalize and endorse stigmatizing views of mood disorders than women [35]; and are unlikely to connect the symptoms they experience to a mood disorder [66]. This lack of recognition or stigmatization of mental health issues in men is problematic [14]. Absent any large paradigm shift in how gender and masculinity are treated and expressed, some researchers have begun to call for more tailored and specific mental health interventions for men [63, 66], which was not included in this study. We found support, however, for promoting help-seeking behaviours in men, seniors and other at-risk groups. It may be that this gender gap in policy support would disappear, or change directions when asked about more tailored policies.

Political alignment is, as hypothesized, a very important explanatory variable for modelling support for all six questions, which supports previous research on partisanship and mental health policy [37]. Using the political centre as the baseline (6 on the 1 to 11 scale), the odds of strongly supporting any of the policies (versus somewhat support or somewhat oppose) increases as respondents move left and decreases as respondents move right. The differences at the extreme ends of the political spectrum are very large, but so are the margins of error because these extreme positions are more sparsely populated compared to the centre, especially among policy influencers. It is worth noting as well that while the odds of support swing very high and very low at the extremes, all of these policies are supported by a strong majority of respondents, regardless of province or sample type.

Respondents who had immigrated to Canada, Indigenous respondents, and those who identified as belonging to a visible minority were also more supportive of mental health policies when compared to their White and Canadian-born peers. On average, individuals who immigrated to Canada had 1.5 times higher odds of strongly supporting nearly all of the mental health policy options (versus somewhat support or somewhat oppose) compared to individuals born in Canada. An expected but interesting result was that immigrants were more supportive of providing information to new immigrants and refugees about mental health and adjustment to Canada even when controlling for political alignment and visible minority identification as a nominal effect. Immigrants to Canada may have a shared value system or perspective resulting from their immigration experience, regardless of their country of origin.

Indigenous respondents also had more than twice the odds of strongly supporting (versus somewhat support or somewhat oppose) “Support First Nations, Métis, and Inuit control of mental health services” compared to their settler counterparts. This aligns with hypotheses that populations would more strongly support policies directly affecting them and may be further explained by settler governments continued failure to meet the mental health needs of Indigenous Peoples [67].

Those with less than a university professional or graduate degree were less likely to strongly support “Provide information to new immigrants and refugees upon arrival about common mental health problems that may occur with adjustment to Canada and available resources” and those who had completed trade school were also more likely to oppose funding housing services and income supports for individuals with mental health issues. These results were found while still controlling for gender and political alignment, and the inclusion of income did not change the coefficient values. Trade school education in particular has a strong culture of masculinity and individualism that may not lend itself to a supportive stance on issues of mental health [68]. Future research may offer greater explanation on the potential interactions between gender, education, and socioeconomic status—particular to the cultural values of blue-collar workers—that are not examined in these simpler explanatory models here.

Those with lower self-rated mental health were more supportive of policies compared to those who had excellent self-rated mental health, but only for two of the policy questions: “Mandate curricula/training related to mental health promotion, anti-stigma awareness, and suicide prevention among healthcare professionals”, and “Implement a school-based prevention programming that incorporates curricula on suicide and related issues (e.g., anxiety-prevention, resiliency-building, socio-emotional health) and expand workshops and peer support programs in schools”. This partially supports our research hypothesis that those with poorer mental health would be more supportive of these policies, given that they ostensibly stand to gain the most from such policies. The support for policy and programs based on education and training may reflect first-hand experiences of those who may have interacted with mental health supports in the health care system, and demonstrate a desire for improved quality of care through such training. These somewhat disparate results may align with other research on how those with living with depression in particular tend to have lower civic engagement and disenfranchisement from the democratic process [20, 69]; or the effects of the stigmatization of certain mental health experiences (i.e. schizophrenia, antisocial personality disorder) more than the increasingly normalized mood disorders [22, 70], although there is a dearth of research in this area.

### Strengths and limitations

While understandable, the low response rate is a limitation of our study, which has implications for generalizability. The small sample size of policy influencers limited possible inferences because we could not use multiple imputation or modelling on the policy questions that were asked exclusively of the policy influencer sample. We validated all assumptions possible, but some were not possible, particularly around the multiple imputation and missing data processes. The data driven approach permitted rigorous model building, but may have missed some nuances that only empirical research can provide. Using the graphical methods to validate the proportional odds assumption is best practice, however is not an exact science. It is possible that for some variables, the cut off points are different at different levels and may invalidate this assumption. Further, the sample of respondents who selected ‘gender diverse’ or ‘gender—other’ was too small to analyse.

A strength of our paper is that it offers novel research on public and policymaker opinion regarding mental health policy, in contrast to much of the literature which focuses on general public views of people with mental disorders. This research helps to meet the need for understanding support for healthy public policy, related to mental health. We addressed this gap and provided a foundation for future work in this area while also supporting the work of other researchers. Here we were also successful in our use of multiple imputation techniques, allowing for stronger inference despite missing data. The use of modelling techniques to examine individual sub-groups while controlling for other variables permitted examination of potential effect of these covariates in isolation. No important differences were found between provinces, indicating generalizability of the results within a western Canadian context, with important implications for policy practitioners and advocates interested in advancing mental health policy in their jurisdictions.

## Conclusions

The mental health policies explored here have strong support across the general public and policy influencer samples, and across provinces. This support combined with their desirable non-invasiveness as defined using the NCB Intervention Ladder, can help mental health advocates to continue to push for the development and implementation of these policies, especially those that may seem more controversial such as housing and income supports. This research also provides more evidence that men need to be targeted more directly in advocacy, likely through targeted awareness and education campaigns, to try and underscore the importance of mental health and reduce stigma. Additionally, advocacy groups should continue to promote policy change in a non-partisan fashion to avoid deepening the divide in support between right and left. Our novel research shows that mental health policy is well supported, and this creates opportunity to advocate for greater prioritization of mental health policy in Canada.

## Data Availability

The datasets analysed during the current study are available from the corresponding author on reasonable request.

## Abbreviations

CDPS: Chronic Disease Prevention Survey
DALY: Disability adjusted life years
GDP: Gross domestic product
LGBTQ2S +: Lesbian, gay, bisexual, transgender, queer or questioning, two spirit, plus
NCB: Nuffield Council on Bioethics
NIMBY: Not in my back yard
YLD: Years lived with disability
